# Optimal Cut-off Points of Fasting and Post-Glucose Stimulus Surrogates of Insulin Resistance as Predictors of Metabolic Syndrome in Adolescents According to Several Definitions

**DOI:** 10.4274/jcrpe.4873

**Published:** 2018-05-18

**Authors:** Mónica Ivette Piña-Aguero, Aranza Zaldivar-Delgado, Alejandra Salas-Fernández, Azucena Martínez-Basila, Mariela Bernabe-Garcia, Jorge Maldonado-Hernández

**Affiliations:** 1 Instituto Mexicano del Seguro Social (IMSS), National Medical Center Siglo XXI, Pediatrics Hospital, Unit of Medical Research in Nutrition, Mexico City, Mexico

**Keywords:** Insulin resistance surrogates, oral glucose tolerance test, HOMA-IR, cut-off points, metabolic syndrome, adolescents

## Abstract

**Objective:**

The aim of this study was to determine optimal cut-off points for fasting and post-glucose stimulus surrogates of insulin resistance to predict metabolic syndrome in adolescents according to several definitions.

**Methods:**

One hundred fifty-five adolescents living in Mexico City were enrolled during 2011 and 2012. Waist circumference and blood pressure were recorded. Subjects received an oral glucose load of 1.75 g per kg up to a maximum dose of 75 g. Blood samples were drawn at baseline and 120 minutes. Concentrations of plasma glucose, triglycerides, high-density lipoprotein cholesterol and insulin were determined.

**Results:**

The frequency of metabolic syndrome showed a large variability when using a variety of published definitions; in contrast, the optimal cut-off points for fasting insulin, homeostatic model assessment of insulin resistance and two-hour oral glucose tolerance test insulin were very similar in almost all the definitions considered and had adequate diagnostic performance: area under the curve >0.869, sensitivity >0.835 and specificity >0.755. Insulin resistance surrogates had substantial agreements with Ford, Cook and Salas definitions (Kappa~0.62; agreement~82%); moderate agreement was observed for International Diabetes Federation, Cruz and Ferranti definitions (Kappa~0.41–0.59; agreement~77%).

**Conclusions:**

Insulin resistance surrogates may be a better approach for metabolic syndrome assessment in an adolescent population because of reduced variability and a higher predictive value.

## What is already known on this topic?

There is no consensus for defining metabolic syndrome in the pediatric population. More than 40 different definitions for this population have been published to date. It is difficult to determine which definition is the most appropriate for clinical settings because of the variability of the prevalence reported.

## What this study adds?

The use of an insulin resistance surrogate could be an adequate strategy to unify the diversity of diagnostic criteria. We are proposing, as surrogates of insulin resistance, cut-off points for post-stimulus insulin and glucose concentrations in a pediatric population.

## Introduction

In 1988, Reaven (1) coined the term “Syndrome X” for the cluster of clinical features: insulin resistance (IR), hypertension, raised triglycerides (TG) and decreased high-density lipoprotein-cholesterol (HDL-C). These individual criteria often co-occur and increase the risk of coronary artery disease. One decade later, in 1998, the World Health Organization (WHO) proposed a global definition for metabolic syndrome (MS) that consisted of impaired glucose tolerance or diabetes mellitus and/or IR determined by hyperinsulinemic-euglycemic clamp and two or more of the following components: elevated arterial pressure (≥140/90 mmHg), raised plasma TG, low HDL-C, microalbuminuria and, for the first time, central obesity (2). However, one year later, B. Balkau and M.A. Charles of the European Group for the Study of Insulin Resistance reviewed the WHO definition of MS. They advised to designate it as “IR syndrome” recognizing the importance of IR in its etiology and proposed to dispense with the hyperinsulinemic-euglycemic clamp and replace it by a surrogate of IR, that might be less invasive and more appropriate for epidemiological and clinical situations (3). Some years later, the National Cholesterol Education Program, developed one of the most widely accepted definitions (4) and finally, the International Diabetes Federation (IDF) proposed another in 2005, which has been quite controversial for use in pediatric population (5). Currently, MS in a pediatric population is defined as the combined and simultaneous presence of three or more of the following criteria: abdominal obesity, dyslipidemia (increased TG and/or decreased HDL-C), metabolic glucose impairment, and/or elevated blood pressure (BP) (5). 

Nowadays there is no a standard, globally-accepted definition for MS in pediatric patients; although more than 40 sets of suggested criteria (definitions) have been published for this population to date. Golley et al (6) reported a prevalence variation of MS from 0 to 59% using six different definitions in a single population of pre-pubertal overweight children. Consequently, it is extremely difficult to determine which of them might be the most appropriate for the clinical setting (7).

Although initially IR was closely linked to MS, recent definitions do not consider a surrogate for IR as a formal component of it. Instead, fasting glucose concentrations are used as a marker of alterations in glucose metabolism, notwithstanding this usually manifests later in the natural history of the disease. Some authors have discussed the lack of sensitivity of fasting glucose for detecting impaired glucose tolerance (8,9,10). 

The use of an IR surrogate could be a strategy to unify the diversity of diagnostic criteria published currently, reduce prevalence variability among populations and allow the identification of subjects at risk with a high predictive level. Therefore, the aim of this study was: 1) to determine optimal cut-off points of fasting and post-glucose stimulus IR surrogates to predict MS in adolescents, according to several definitions published in the pediatric population and; 2) to estimate the level of agreement between the analyzed definitions and the IR surrogates’ cut-off points suggested.

## Methods

### Study Population

The data from this cross-sectional study correspond to two previous studies published by our group (11,12). One hundred fifty-five apparently healthy adolescents aged between 10 and 18 years from an open population living in Mexico City were enrolled during 2011 and 2012. The study protocol complied with the World Medical Association Declaration of Helsinki regarding ethical conduct of research in human subjects. The study protocol was approved by the Ethics Committee of the Instituto Mexicano del Seguro Social (registered as R-2010-3603-35). All subjects assented to participate in the study and informed consent was provided by their parents. Subjects with current chronic disease, such as type 2 diabetes mellitus, or using medications that affect glucose metabolism or a history of fever in the last 48 hours, were excluded. To avoid possible complications during the oral glucose tolerance test (OGTT), pre-study screening was carried out and patients with capillary blood glucose ≥126 mg/dL were not included.

### Study Protocol

Voluntary participants arrived at the Unit of Medical Research in Nutrition with their parents or legal guardians at 8:00 am after an eight hour fast. Weight and height were recorded with light clothing and without shoes. Weight was assessed to the nearest 0.1 kg with a standard scale by using a fixed balance (Tanita, Arlington Heights, IL, TBF-300A) and height was measured to the nearest 0.1 cm using a wall stadiometer. Body mass index was calculated by dividing body weight (kg) by height squared (m^2^). Waist circumference was determined with a non-elastic tape to the nearest millimeter at the midpoint between the lowest rib margin and the iliac crest, at the end of a gentle expiration. All measurements were obtained with the subject in a standing position. BP was measured in the right arm after a resting period of five minutes, while subjects were seated properly, as described by the National Heart, Lung, and Blood Institute (NHLBI) (13). Subjects received an oral glucose load of 1.75 g per kg of body weight up to a maximum dose of 75 g (ACS reagent; Sigma-Aldrich, St. Louis, MO), dissolved in 150 mL of water for the OGTT. Blood samples were drawn at baseline and 120 minutes through an antecubital venipuncture into Vacutainer test tubes with ethylenediaminetetraacetic acid. Samples were centrifuged at 3000 rpm for 10 minutes. Plasma was preserved at -70 °C until analysis.

### Blood Analysis

Concentrations of plasma glucose, TG and HDL-C were determined with commercial kits in a Spin 120 automated spectrophotometer (Spinreact, Girona, Spain; coefficients of variation ~3.9%). Insulin was determined by commercial radioimmunoassay kits (Millipore, Billerica, MA) with coefficient of variation of 7.5%. Homeostatic model assessment of insulin resistance (HOMA-IR) was calculated with the following formula (14):

HOMA-IR=[fasting insulin (μU/mL)*fasting glucose (mg/dL)] / 405

### Metabolic Syndrome Definitions

MS was assessed according to six different definitions: Cook et al (2003) (15), Cruz et al (2004) (16), de Ferranti et al (2004) (17), Ford et al (2005) (18), IDF (2007) (5) and Salas-Fernández et al (2015) (12). Definitions are summarized in Table 1

The fourth report for the diagnosis, evaluation, and treatment of high BP in children and adolescents of the NHLBI (13) was used to establish elevated arterial pressure in the participants of our study. Waist circumference was assessed using the reference percentiles published by Fernández et al (19). Elevated plasma TG and low HDL-C for Cruz et al (16) definition were assessed against the reference tables from Hickman et al (20).

### Statistical Analysis

Data analyses were performed with IBM SPSS Statistics for Windows software (SPSS version 22.0; IBM Corp., Armonk, NY). Kolmogorov-Smirnov test was used to assess data normality. Data are presented as median (range) as they were shown to be nonparametric. Mann-Whitney U test was used for inter-groups comparison according to gender. Several receiver operator characteristic (ROC) curves were constructed to determine optimal cut-off points for different IR surrogates to predict MS according to several definitions. ROC curves were computed by comparison to data from healthy subjects (exhibiting no components of MS) versus subjects with a high risk of MS (two components) and those with MS (three or more components), according to each definition. Area under the curve with 95% confidence interval was obtained; positive and negative predictive values were determined (positive predictive value and negative predictive value respectively). IR surrogates’ cut-off points were selected according to performance for MS assessment on the ROC curve analysis. Those with higher levels of sensitivity and specificity were adopted for the purpose of this study. To assess agreement between the estimated cut-off points for IR surrogates and definitions of MS, a kappa coefficient was computed and percentage of agreement is also displayed.

## Results

A total of 155 adolescents living in Mexico City were enrolled during 2011 and 2012 (83 males and 72 females). Clinical and metabolic characteristics of subjects are summarized in Table 2. Median age was 12.9 years for males and 13.6 for females. There was no statistical difference between most of the biochemical and clinical variables when genders were compared. However, females had significantly higher levels of TG and lower concentrations of fasting glucose. Simple regression analyses were used to determine the influence of gender and Tanner stage on fasting insulin (b=1.4; p=0.27 and b= -1.2; p=0.14, respectively), HOMA-IR (b=0.11; p=0.71 and b= -0.4; p=0.03, respectively) and 2-hour insulin after an OGTT (b=11; p=0.10 and b=-5.8; p=0.17). Since no substantial effects were observed in IR surrogates, subsequent analyses were not stratified by gender or Tanner stage.

**Figure 1:**
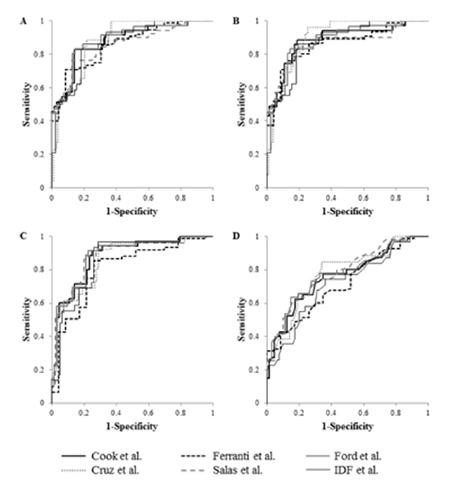
Performance of insulin resistance surrogates for metabolic syndrome assessment. Receiver operating characteristic curves evaluating the sensitivity and specificity of A) Fasting insulin, B) homeostatic model assessment of insulin resistance, C) 2 hours oral glucose tolerance test insulin and D) 2 hours oral glucose tolerance test glucose for assessment of metabolic syndrome according to different definitions

The frequency of MS in the studied population showed large variability across different definitions; Cook and Ford had a similar frequency of 11% and 11.6% respectively, Ferranti 29.7%, Salas 19.4%, Cruz 4.5% and IDF 3.2%. In contrast, the optimal cut-off points for IR surrogates were very similar in almost all the studied definitions (Table 3). Only Ferranti’s data partially disagreed. Furthermore, IR surrogates presented a high predictive level for MS, regardless of the definition used, as shown in Figure 1. Average fasting insulin cut-off point had a sensitivity and specificity of 0.835 and 0.808, respectively. Very similar values were found for HOMA-IR and 2 h OGTT insulin. Lower levels of sensitivity and specificity were observed for 2 h OGTT glucose (0.735 and 0.694, respectively). 

Finally, Kappa coefficients (K) and percentage of agreement between the average cut-off points for IR surrogates and different definitions of MS are shown in table 4. IR surrogates had substantial agreements with the Ford, Salas and Cook definitions (K~0.62; agreement~82%); moderate agreement was observed for IDF, Cruz and Ferranti definitions (K~0.41-0.59; agreement~77%). In terms of agreement between IR surrogates, 2 h OGTT glucose showed the weakest match value to the other surrogates (K~0.18-0.43; agreement~69%).

## Discussion

As expected, considerable variation of MS frequencies was found in the studied population using the different definitions we considered. The highest frequency was observed with Ferranti´s definition (29.7%) and the lowest with the IDF criteria (3.2%). This variability in MS frequencies is due to the diversity of cut-off points used by each author for the different components of MS. Ferranti’s definition, for example, uses a lower threshold for waist circumference (75^th^ percentile) in comparison with the other definitions (90^th^ percentile). As a result, the number of subjects with central obesity is increased in Ferranti’s definition, which in turn has a direct effect on higher prevalence. Conversely, IDF definition designated higher thresholds for elevated TG (>150 mg/dL) and raised BP (≥130/85 mmHg). According to the NHLBI reference tables (13), the 90^th^ percentile for a girl or a boy of 12-years-old and height percentile >95^th^, corresponds to a value of 122-123 mmHg for systolic BP and 78-79 for diastolic BP, respectively. Therefore, from a physiological point of view, the BP threshold proposed by the IDF seems to be rarely found under normal conditions and could underestimate the presence of alterations on it. Finally, as in the IDF definition, Cruz et al (16) propose a higher cut-off points for serum TG (135 and 170 mg/dL for boys and girls between 12 and 15 years-old, respectively). The lower frequencies observed with these definitions are partially due to the low likelihood of exceeding these thresholds at an early age. 

Independently of the variation of the frequencies observed among the MS definitions used in this study, IR surrogates seem to show more consistency. Proposed optimal cut-off points for fasting plasma insulin, HOMA-IR and 2 h OGTT insulin are almost the same when different definitions are compared (Figure 1). Only Ferranti’s definition showed discrepant values and for this reason it was excluded from an average cut-off point that was computed for each IR surrogate: 14.38 µU/mL for fasting insulin, 2.97 for HOMA-IR and 45.68 µU/mL for 2h-OGTT insulin. Additionally, the suggested cut-off points showed an adequate diagnostic performance, as denoted by the diagnostic attributes summarized in Table 3 and displayed a substantial agreement across three of the definitions: Ford et al (18), Cook et al (15) and Salas-Fernández et al (12) (Table 4). As a whole, these data support that the use of an IR surrogate could be an adequate strategy to unify the diversity of diagnostic criterion for MS assessment published at present. In contrast, the proposed cut-off point for 2h-OGTT glucose (98.37 mg/dL) is not valid for MS assessment because it showed a poorer diagnostic performance and lower kappa coefficient and percentage of agreement (Table 3 and 4). It is well known that fasting and post-stimulus plasma glucose is not an adequate surrogate of IR, as it remains in the normal range due to the hyperinsulinemia that characterizes this alteration. Raised fasting and post-stimulus glucose levels (≥100 and ≥140 mg/dL, respectively) (21) are indicators of an advanced metabolic impairment (glucose intolerance) in which insulin can no longer maintain blood glucose levels in the normal range. Instead IR surrogates unmask an early alteration in glucose metabolism, which makes them more suitable for MS assessment. 

The MS definitions used in this manuscript derive from a variety of studies that were performed in different population types and sample sizes. Data for Cook and Ferranti’s definitions were extracted from the Third National Health and Nutrition Examination Survey (NHANES III; 1988-1994) using 2430 and 1960 children aged 12 to 19 years, respectively. Ford used data from 1366 participants aged 12-17 years from the NHANES (1999-2000). Cruz and Salas’s definitions were in smaller samples of Hispanic children (n=126; 8-13 years living in Los angeles, California and n=155; 10-18 years from Mexico City, respectively). Regardless of these important differences, the estimated cut-off points for fasting insulin, HOMA-IR and 2h-OGTT insulin are remarkably homogeneous among the different populations used to create the definitions analyzed, so they could be extrapolated for similar populations.

### Study Limitations

It is important to note that a disadvantage in the use of fasting and post-stimulus IR surrogates is the high within-subject variability reported previously by other authors. Reinehr et al (22) reported a coefficient of variation (CV) of 22% for HOMA-IR in children and adolescents. In Mexican adults, our group found similar CV for fasting insulin and HOMA-IR (20.7% and 19.3%, respectively) and for 2h-OGTT insulin a CV of 29.9% was observed (23). Additionally, Schousboe et al (24) found a CV of 54% for 2h-OGTT insulin in a population of Danish origin. Since fasting IR surrogates seem to have lower intrasubject variability, these could be a better alternative for MS assessment. Furthermore HOMA-IR has proved to be an adequate tool in clinical and epidemiological studies. According to a revision carried out by Wallace et al (25) this surrogate had shown a good correlation when compared with the euglycemic clamp (r=0.58-0.88, p<0.0001).

A strength of this study is that we are proposing cut-off points for post-stimulus insulin and glucose concentrations. Due to the scarcity of information related to this, we consider that this is a valuable contribution of this work.

## Conclusion

Currently there is no standard definition for MS in pediatrics. This leads to underestimation or overestimation of its prevalence, severely limits the comparability of different studies and compromises its usefulness in the clinical setting. The use of an IR surrogate may be a better approach to assess subjects at risk of MS in pediatric populations as the IR surrogates used in this study exhibit less variability and a high predictive level.

## Figures and Tables

**Table 1:**
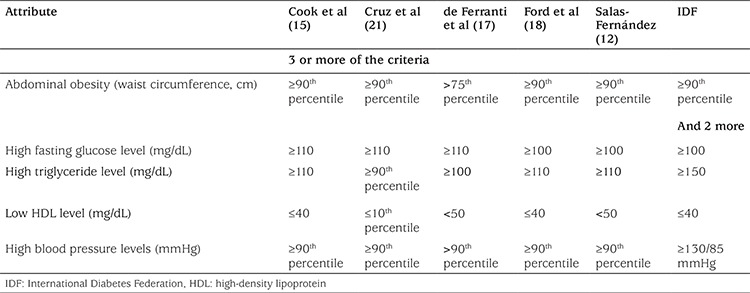
Metabolic syndrome definitions

**Table 2:**
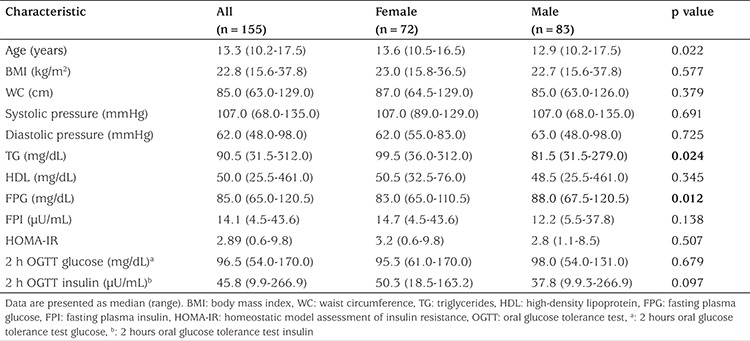
Clinical and metabolic characteristics of study subjects

**Table 3:**
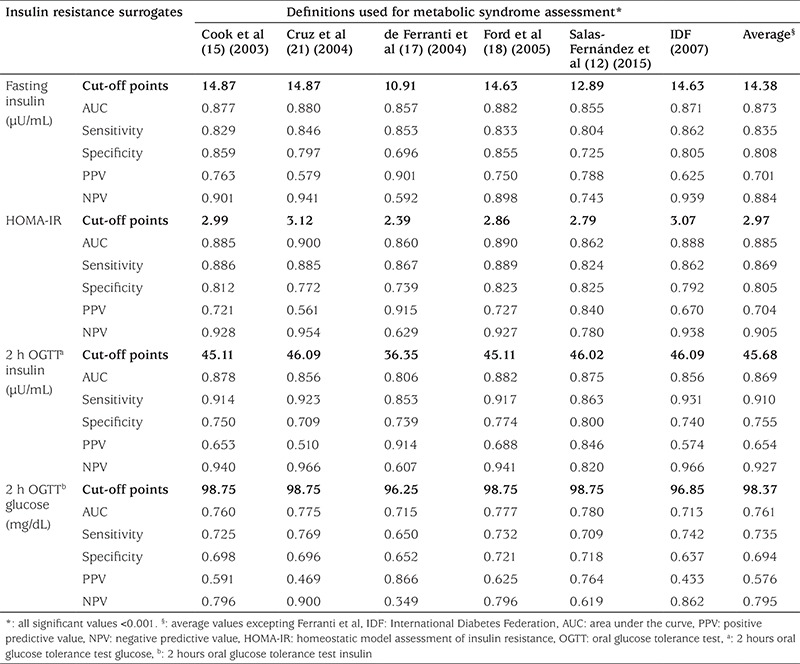
Cut-off points for metabolic syndrome assessment in adolescents according to different definitions

**Table 4:**
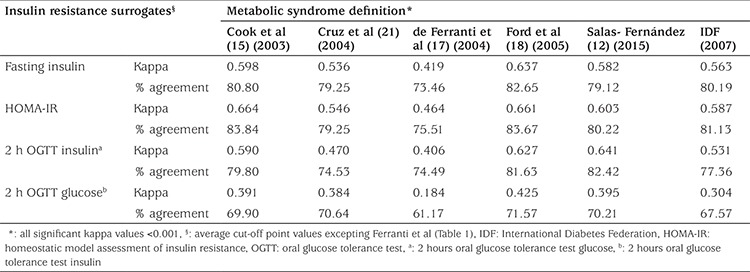
Agreement between insulin resistance surrogates and metabolic syndrome definitions
